# Prescribing patterns of encounters in fourteen general practice clinics in rural Beijing: a cross-sectional study

**DOI:** 10.1186/s12913-019-4656-2

**Published:** 2019-11-06

**Authors:** Guanghui Jin, Chao Chen, Yanli Liu, Yali Zhao, Lifen Chen, Juan Du, Xiaoqin Lu, Jianjun Chen

**Affiliations:** 1 Department of General Practice, School of General Practice and Continuing Education, Capital Medical University, Beijing, China; 2 Department of Education, Xuanwu Hospital, Capital Medical University, Beijing, China; 3 Department of General Practice, Beijing Tiantan Hospital, Capital Medical University, Beijing, China; 4 Department of Education, Beijing Friendship Hospital, Capital Medical University, Beijing, China

**Keywords:** Prescribing patterns, Medicines, General practice, Community health service, Rural, Beijing

## Abstract

**Background:**

General practice clinics are the main primary care institutions providing ambulatory care in the rural areas of Beijing, rational use of medicines is crucial for the rural primary care system. This study investigated the prescribing patterns of general practice clinics in rural Beijing to provide a baseline for monitoring and promoting the rational use of medicines.

**Methods:**

We performed a cross-sectional study at 14 rural community health service centers in 6 non-central districts of Beijing sampled through a multistage approach, 85 general practitioners were selected from the 14 centers. Total 8500 prescriptions were derived by recording 100 consecutive patients of each the general practitioner. The World Health Organization drug use indicators and an additional indicator were adopted to assess the prescribing patterns.

**Results:**

The median number of medicines per encounter was 2.0 (1.0, 2.0); the percentage of generics and essential medicines prescribed were 97.0 and 58.2%, respectively; the percentage of encounters with antibiotics prescribed was 15.1%; the percentage of encounters with injections prescribed was 3.7%; the percentage of encounters with traditional Chinese patent medicines prescribed was 52.5%; the median duration of consultation time was 6.0 (4.0, 10.0) minutes. The most frequently prescribed medicine was aspirin (low dose, 4.6%). The prescribing indicators were influenced by different patient characteristics, patients with new cooperative rural medical scheme were less likely to be prescribed with ≥3 medicines (OR 0.865), essential medicines (OR 0.812) and traditional Chinese patent medicines (OR 0.631), but were more likely to be prescribed with injections (OR 1.551) in the encounter. Patients with ≥3 problems were more likely to be prescribed with ≥3 medicines (OR 6.753), antibiotics (OR 2.875) and traditional Chinese patent medicines (OR 2.926) in the encounter.

**Conclusions:**

Most indicators in this study showed similar or fair performance in comparison with World Health Organization and domestic reports, except the percentage of medicines prescribed from the essential medicine list. Regular monitoring on the prescription quality of general practice clinics in rural Beijing should be maintained.

## Background

In 1985, the World Health Organization (WHO) convened a conference on the rational use of medicines. In the ensuing report, rational use of medicines was defined as ‘patients receive medicines appropriate to their clinical needs, in doses that meet their own individual requirements for an adequate period of time, at the lowest cost to them and their community’ [1]. Irrational use of medicines is a global problem, it is essential to have reliable data to provide benchmark and monitor the pattern of how medicines are used, make comparisons among countries, regions, facilities and identify problems to develop intervention strategies [2]. The WHO had defined a number of quality indicators for investigating prescribing patterns allowing stakeholders and researchers to make comparisons between situations in different regions and different periods [3].

Primary care has become a very important focus of health care reform in China, the government intends to establish a tiered health care system and strengthen the role of primary care as the first contact of health care system [4]. Community health service institutions (CHSIs), including community health service centers (CHSCs) and community health service stations (CHSSs), are the main primary care institutions which provide medical care, preventive care, health promotion, rehabilitation, health education and family planning in the community [5]. A CHSC usually consists of general practice clinic, traditional Chinese medicine clinic, preventive care, women and children health, laboratory tests, pharmacy etc. General practice clinics in CHSCs undertake the main responsibility in delivering ambulatory care in the community. To ensure the accessibility and rational use of medicines in primary care, a series of policies had been implemented in CHSIs, including the establishment of essential medicine system [6] and the zero mark-up medicine regulation in 2009 [7], as well as the campaign to promote rational use of antibiotics since 2012 [8]. In the rural areas, a tiered health care system was built as well, including county hospitals (usually secondary care hospitals), CHSCs and village clinics [9]. The rural primary care system comprises the CHSCs and village clinics, and primary care doctors including general practitioners (GPs) and village doctors are the main prescribers in the rural areas [10, 11]. CHSCs are the key component of rural primary care network, providing primary medical care and public health services, coordinating care between county hospitals and village clinics.

As the capital of China, Beijing has established a relatively sophisticated community health service network, there were 326 CHSCs and 11,276 GPs till the end of 2014 in Beijing [12]. Beijing has a rural population of 2,933,000 people with an annual income of 20,226 RMB (43,910 RMB for the urban population) in 2014, and rural residents spent 6.4% of their income on out of pocket payment in 2013 [13]. General practice clinics in rural Beijing play the main role in providing ambulatory care as well, it is very important to ensure the access to medicines in the rural areas due to relatively lower income and limited health resources. Policies to promote the accessibility and rational use of medicines had been carried out in the rural areas of Beijing, but the prescribing patterns of general practice clinics have not been assessed yet. Thus, the aim of the study is to utilize the WHO drug use indicators as well as an additional indicator to describe the prescribing patterns of general practice clinics in CHSCs in rural Beijing to provide a baseline for monitoring and promoting the rational use of medicines.

## Methods

### Study design

A cross-sectional study was carried out to describe the prescribing patterns of general practice clinics in 14 CHSCs by the WHO drug use indicators and an additional indicator in the rural areas of Beijing.

### Setting and participants

The WHO’s guideline on drug use study recommends 20 facilities for a cross-sectional survey, and at least 30 encounters per facility, which entails at least 600 encounters in total to describe drug use patterns [3]. There are 16 districts in Beijing including 6 central urban districts and 10 non-central districts mainly comprise suburban and rural areas, according to the Guideline on the Division of Urban and Rural Areas from the National Bureau of Statistics of China [14]. We employed a multistage sampling approach, in the first stage, 6 out of 10 non-central districts in Beijing were chosen randomly. In the second stage, 14 rural public CHSCs in the 6 non-central districts were selected by purposive sampling based on the following criteria: (1) location in the rural areas; (2) stable amount of patients; (3) availability for the study. In the third stage, we recruited 85 GPs who met the following criteria by purposive sampling as well: (1) work experience in CHSC for 5 years or above; (2) possession of the medical license of practicing doctor; (3) possession of general practice postgraduate training certificate; (4) undertaking clinical work in general practice clinic; (5) availability for the study. The GPs’ 100 consecutive encounters were recorded. We selected fewer than 20 facilities in this study, but the CHSCs are very standardized institutions given their public nature, which would improve the representativeness of the sample.

### Prescribing indicators

Six core drug use indicators proposed by the WHO were used to assess the prescribing patterns [3], including: (1) the median number of medicines per encounter; (2) the percentage of medicines prescribed by generic name; (3) the percentage of encounters with antibiotics prescribed; (4) the percentage of encounters with injections prescribed; (5) the percentage of medicines prescribed from China’s National Essential Medicines List (EML) of 2012; (6) the median duration of consultation time. In addition, the percentage of encounters with traditional Chinese patent medicines (TCPMs) prescribed was also included as an indicator given the large amount of TCPMs prescribed in general practice clinics. TCPM uses traditional Chinese medicines and herbs as raw materials and refines the materials into various dosage forms such as dripping pills (small ball-shaped pills), liquids, powders, capsules, etc. [15].

### Field work

A research panel consisted of two general practice researchers, two GPs and three general practice postgraduate students was set up. A data collection form was developed on the basis of literature review and discussion. Ten assistants who were trainees in the CHSCs were recruited to help the GPs record the information of 100 consecutive encounters during or after the consultation. Training on the purpose and protocols of the study was delivered to the participating GPs and the assistants before the investigation. We also assured the GPs that the data would only be used for research, their personal information could not be traced, to minimize the influence on their prescribing behavior. The postgraduate students were responsible for monitoring the process of study and reporting to the general practice researchers. The field work lasted from December 8, 2014 to January 27, 2015.

### Statistical analyses

EpiData (Version 3.1, EpiData Association, Odense, Denmark) was used to set up the database and double-entry was performed to control data entry errors. All data analyses were carried out using Statistical Package for Social Science (SPSS) for Microsoft Windows (Version 17.0, SPSS Inc., Chicago, IL, USA). Descriptive statistics (median, interquartile range, percentage) were employed to describe the demographic information of GPs, encounter characteristics and prescribing indicators. The differences in prescribing patterns were analyzed by Mann-Whitney U test (for comparing medians between two groups), Kruskal-Wallis test (for comparing medians among over two groups) and Chi-square test (for comparing percentages). Binary logistic regression was performed to analyze the influence of patient characteristics as 9 independent variables on 6 prescribing indicators as dependent variables, encounters with missing values were excluded in the logistic regression analysis, total 7910 encounters were analyzed, the results were expressed as odds ratios (OR) with 95% confidence intervals (CI). The level of significance was set at *P* ≤ 0.05.

## Results

### Demographics of the GPs and characteristics of the encounters

Total 8500 encounters comprised 100 consecutive patients of each GP were recorded. More female GPs (62.4%) participated in the study, most GPs (60.0%) were between 36 and 45 years old, and had been working for 11–20 years (40.0%) or beyond (48.2%).

Over half (57.9%) of the patients presented with at least one symptom, the second most frequent reason for the encounters was prescription refill without other complaint (29.6%). Most patients were with 3 insurance schemes including public institution insurance scheme (PIIS, 24.8%), employee basic medical scheme (EBMS, 24.7%) and the new rural cooperative medical scheme (NRCMS, 40.7%). PIIS is designed for the employees of public institutions e.g. public education or government institutions, PIIS has the best reimbursement rate and more extensive medicine coverage, and NRCMS is designed for the rural areas. 60.8% of the encounters were contracted patients who had signed a contract with a GP to establish a long-term relationship. Demographics of the GPs and characteristics of the encounters were presented in Tables 1 and 2.

**Table 1:** Demographics of the GPs (*n* = 85)

Demographics of the GPs	n (percentage)
Gender	
Male	32 (37.6%)
Female	53 (62.4%)
Age group	
≤ 35 years old	19 (22.4%)
36–45 years old	51 (60.0%)
≥ 46 years old	15 (17.6%)
Work experience	
5–10 years	10 (11.8%)
11–20 years	34 (40.0%)
> 20 years	41 (48.2%)

*Abbreviation*: *GP* General practitioner

**Table 2:** Characteristics of the encounters (*n* = 8500)

Encounter characteristics	n (percentage)
Gender	
Male	4046 (47.6%)
Female	4436 (52.2%)
Missing	18 (0.2%)
Age group	
0–24 years old	355 (4.2%)
25–44 years old	1551 (18.2%)
45–59 years old	3095 (36.4%)
≥ 60 years old	3456 (40.7%)
Missing	43 (0.5%)
Reason for encounter	
Present with at least one symptom	4920 (57.9%)
Regular follow up	769 (9.0%)
Prescription refill without other complaint	2513 (29.6%)
Other reasons	298 (3.5%)
Insurance type	
PIIS	2111 (24.8%)
EBMS	2096 (24.7%)
NRCMS	3463 (40.7%)
Other insurance	300 (3.5%)
No insurance	431 (5.1%)
Missing	99 (1.2%)
First time visit	
Yes	521 (6.1%)
No	7750 (91.2%)
Missing	229 (2.7%)
Contracted patient	
Yes	5167 (60.8%)
No	3074 (36.2%)
Missing	259 (3.0%)
Chronic disease	
Yes	4902 (57.7%)
No	3598 (42.3%)
Number of problems discussed	
< 3	7323 (86.2%)
≥ 3	1177 (13.8%)
Consultation time	
< 5 min	2774 (32.6%)
5–10 min	3778 (44.4%)
> 10 min	1721 (20.3%)
Missing	227 (2.7%)

*Abbreviation*: *PIIS* Public institution insurance scheme, *EBMS* Employee basic medical scheme, *NRCMS* New rural cooperative medical scheme

Other reasons for encounter included check-up, transfusion and injection, sickness certificate, etc.

### Prescribing indicators of the encounters

Of the 8500 encounters, there were 7870 (92.6%) encounters with at least one medicine prescribed. Total 16,067 medicines were prescribed, including 9553 (59.5%) Western medicines and 6514 (40.5%) TCPMs. The median number of medicines per encounter was 2.0 (1.0, 2.0); the percentage of generic medicines prescribed was 97.0%; the percentage of encounters with antibiotics prescribed was 15.1%; the percentage of encounters with injections prescribed was 3.7%; the percentage of medicines prescribed from EML was 58.2%; the percentage of encounters with TCPMs prescribed was 52.5%; the median duration of consultation time was 6.0 (4.0, 10.0) minutes. The most frequently prescribed medicine was aspirin (low dose, 4.6%). Please see Tables 3 and 4.

**Table 3:** Prescribing indicators of the encounters

Prescribing indicators assessed	Total medicines/encounters	Median (interquartile range)/percentage
Median number of medicines per encounter	16,067	2.0 (1.0, 2.0)
Percentage of encounters with antibiotics prescribed	1285	15.1%
Percentage of encounters with injections prescribed	312	3.7%
Percentage of medicines prescribed by generic name	15,577	97.0%
Percentage of medicines from EML	9343	58.2%
Percentage of encounters with TCPMs prescribed	4459	52.5%
Median duration of consultation time (minutes)	8273	6.0 (4.0, 10.0)

*Abbreviation*: *EML* Essential medicine list, *TCPM* Traditional Chinese patent medicine

**Table 4:** Ten most frequently prescribed medicines (*n* = 16,067)

Rank	Medicine	Frequency	Percentage in all medicines	Whether or not EML	Whether or not TCPM
1	Aspirin (low dose)	739	4.6%	Yes	No
2	Ganmaoqingre (mainly for common cold)	517	3.2%	Yes	Yes
3	Nifedipine	502	3.1%	Yes	No
4	Cefuroxime	422	2.6%	Yes	No
5	Amlodipine	410	2.6%	Yes	No
6	Acarbose	302	1.9%	Yes	No
7	Metformin	296	1.8%	Yes	No
8	Levofloxacin	278	1.7%	Yes	No
9	Ambroxol	271	1.7%	Yes	No
10	Qingkailing (mainly for common cold)	258	1.6%	Yes	Yes

*Abbreviation*: *EML* Essential medicine list, *TCPM* Traditional Chinese patent medicine

### Prescribing patterns of the GPs

Differences were seen in the indicators of median medicines per encounter, injections, essential medicines, and TCPMs in the gender and age groups of GPs. Work experience of the GPs lead to the differences in the indicators of median medicines per encounter, injections and essential medicines, please see Table 5.

**Table 5:** Prescribing patterns of the GPs (*n* = 8500)

Demographics of the GPs	Median medicines per encounter (interquartile range)	Percentage of encounters with generic medicines	Percentage of encounters with antibiotics	Percentage of encounters with injections	Percentage of encounters with essential medicines	Percentage of encounters with TCPMs
Gender						
Male	2.0 (1.0, 2.0)	90.9%	14.9%	2.0%	68.0%	49.8%
Female	2.0 (1.0, 3.0)	91.6%	15.3%	4.7%	72.0%	54.1%
Z or χ^2^	−7.902	1.187	0.235	40.508	15.586	14.411
*P* value	**0.000** ^*^	0.276	0.627	**0.000** ^*^	**0.000** ^*^	**0.000** ^*^
Age group						
≤ 35 years old	2.0 (1.0, 2.0)	90.7%	14.0%	1.9%	63.4%	49.7%
36–45 years old	2.0 (1.0, 3.0)	91.5%	15.0%	4.0%	72.9%	53.4%
≥ 46 years old	2.0 (1.0, 2.0)	91.6%	16.8%	4.8%	71.2%	52.8%
*χ*^2^	55.963	1.440	5.182	23.923	61.174	7.716
*P* value	**0.000** ^*^	0.487	0.075	**0.000** ^*^	**0.000** ^*^	**0.021** ^*^
Work experience						
5–10 years	2.0 (1.0, 2.0)	90.3%	13.3%	0.7%	64.1%	51.8%
11–20 years	2.0 (1.0, 2.0)	90.8%	16.1%	4.4%	71.3%	52.1%
> 20 years	2.0 (1.0, 2.0)	92.1%	14.8%	3.8%	71.4%	52.9%
*χ*^2^	17.443	5.631	5.488	30.037	22.204	0.721
*P* value	**0.000** ^*^	0.060	0.064	**0.000** ^*^	**0.000** ^*^	0.697

*Abbreviation*: *GP* General practitioner, *TCPM* Traditional Chinese patent medicine

^*^Significant in bold

### The influence of encounter characteristics on prescribing indicators

Overall, the prescribing indicators varied by different encounter characteristics. However, no significant difference was found in the gender group in the indicators of median medicines per encounter, generic medicines, antibiotics and TCPMs. No significant difference was found in the ‘first-time visit’ group in the indicator of TCPMs. No significant difference was found in the ‘number of problems involved’ and ‘consultation time’ groups in the indicator of injections. Please see Table 6.

**Table 6:** Prescribing patterns among different groups of encounter characteristics (*n* = 8500)

Encounter characteristics	Median medicines per encounter (interquartile range)	Percentage of encounters with generic medicines	Percentage of encounters with antibiotics	Percentage of encounters with injections	Percentage of encounters with essential medicines	Percentage of encounters with TCPMs
Gender						
Male	2.0 (1.0, 3.0)	91.4%	15.1%	4.2%	71.6%	51.4%
Female	2.0 (1.0, 2.0)	91.1%	15.2%	3.2%	69.5%	53.4%
Missing	18					
Z or χ^2^	−1.743	0.008	0.023	5.705	4.296	3.539
*P* value	0.081	0.928	0.880	**0.017** ^*^	**0.038** ^*^	0.060
Age group						
0–14	2.0 (1.0, 2.0)	85.9%	32.4%	5.9%	55.3%	63.5%
15–24	2.0 (1.0, 2.0)	87.6%	33.0%	7.6%	69.7%	62.2%
25–44	2.0 (1.0, 2.0)	89.9%	23.2%	3.7%	67.6%	62.7%
45–59	2.0 (1.0, 2.0)	91.1%	13.5%	2.9%	69.2%	51.3%
≥ 60	2.0 (1.0, 3.0)	92.8%	11.2%	4.1%	73.9%	47.8%
Missing	43					
χ^2^	12.549	22.742	211.431	17.435	47.095	112.701
*P* value	**0.014** ^*^	**0.000** ^*^	**0.000** ^*^	**0.002** ^*^	**0.000** ^*^	**0.000** ^*^
Reason for encounter						
Present with at least one symptom	2.0 (1.0, 3.0)	92.3%	22.9%	4.9%	69.1%	67.1%
Regular follow up	1.0 (1.0, 2.0)	93.9%	3.9%	2.7%	70.2%	23.4%
Prescription refill without other complaint	2.0 (1.0, 2.0)	97.0%	4.3%	1.5%	79.8%	38.0%
Other reasons	0 (0, 0)	22.1%	6.7%	5.0%	15.4%	7.0%
χ^2^	613.625	1921.523	553.674	57.440	544.088	1139.777
*P* value	**0.000** ^*^	**0.000** ^*^	**0.000** ^*^	**0.000** ^*^	**0.000** ^*^	**0.000** ^*^
Insurance type						
PIIS	2.0 (1.0, 3.0)	94.5%	16.6%	2.8%	75.5%	58.6%
EBMS	2.0 (1.0, 3.0)	94.2%	14.1%	3.1%	71.3%	59.2%
NRCMS	2.0 (1.0, 2.0)	88.9%	13.6%	4.0%	68.1%	44.3%
Other insurance	2.0 (1.0, 3.0)	93.3%	17.3%	3.7%	68.7%	57.3%
No insurance	2.0 (1.0, 2.0)	81.9%	23.2%	8.1%	62.6%	52.4%
Missing	99					
χ^2^	116.527	124.972	34.935	31.825	49.315	164.359
*P* value	**0.000** ^*^	**0.000** ^*^	**0.000** ^*^	**0.000** ^*^	**0.000** ^*^	**0.000** ^*^
First time visit						
Yes	2.0 (1.0, 2.0)	81.8%	22.3%	6.0%	59.1%	52.8%
No	2.0 (1.0, 2.0)	92.0%	14.7%	3.5%	71.1%	52.5%
Missing	229					
Z or χ^2^	−4.823	64.990	21.901	8.014	33.663	0.020
*P* value	**0.000** ^*^	**0.000** ^*^	**0.000** ^*^	**0.005** ^*^	**0.000** ^*^	0.888
Contracted patient						
Yes	2.0 (1.0, 3.0)	92.4%	12.6%	3.3%	72.6%	50.2%
No	2.0 (1.0, 2.0)	89.6%	19.2%	4.3%	66.7%	56.1%
Missing	259					
Z or χ^2^	−6.092	20.185	65.265	5.847	32.177	26.959
*P* value	**0.000** ^*^	**0.000** ^*^	**0.000** ^*^	**0.016** ^*^	**0.000** ^*^	**0.000** ^*^
Chronic disease						
Yes	2.0 (1.0, 3.0)	93.2%	9.3%	3.1%	75.3%	43.3%
No	2.0 (1.0, 2.0)	88.8%	23.0%	4.4%	63.9%	65.0%
Z or χ^2^	−7.330	52.042	305.195	9.166	128.968	395.725
*P* value	**0.000** ^*^	**0.000** ^*^	**0.000** ^*^	**0.002** ^*^	**0.000** ^*^	**0.000** ^*^
Number of problems involved						
< 3	2.0 (1.0, 2.0)	90.5%	14.0%	3.8%	68.1%	49.8%
≥ 3	3.0 (2.0, 4.0)	96.8%	22.3%	3.1%	85.0%	69.0%
Z or χ^2^	−29.714	50.620	54.311	1.447	139.293	149.682
*P* value	**0.000** ^*^	**0.000** ^*^	**0.000** ^*^	0.229	**0.000** ^*^	**0.000** ^*^
Consultation time						
< 5 min	2.0 (1.0, 2.0)	85.9%	13.6%	4.4%	64.2%	48.6%
5–10 min	2.0 (1.0, 3.0)	93.9%	15.9%	3.5%	75.6%	54.9%
> 10 min	2.0 (1.0, 3.0)	95.3%	17.0%	3.2%	71.3%	54.7%
Missing	227					
χ^2^	160.473	171.193	11.100	5.549	99.659	28.615
*P* value	**0.000** ^*^	**0.000** ^*^	**0.004** ^*^	0.062	**0.000** ^*^	**0.000** ^*^

*Abbreviation*: *TCPM* Traditional Chinese patent medicine, *PIIS* Public institution insurance scheme, *EBMS* Employee basic medical scheme, *NRCMS* New rural cooperative medical scheme

Note: other reasons for encounter included check-up, transfusion and injection, sickness certificate, etc.

^*^Significant in bold

In the binary logistic regression analysis, number of medicines prescribed in the encounter was categorized into < 3 medicines and ≥ 3 medicines as a dependent variable, ≥3 medicines were more likely to be prescribed in encounters with male patients (OR 1.138), EBMS (OR 1.279), contracted patients (OR 1.159),≥3 problems (OR 6.753), and consultation time between 5 and 10 min (OR 1.537), but were less likely to be prescribed with older patients, and NRCMS (OR 0.865). Generic medicines were more likely to be prescribed in encounters with prescription refill (OR 2.948), ≥3 problems (OR 2.074), and longer consultation time, but less likely to be prescribed with NRCMS and no insurance and first time visit. Antibiotics were more likely to be prescribed in encounters with ≥3 problems (OR 2.875), but less likely to be prescribed with older patients, and chronic patients (OR 0.455). Injections were more likely to be prescribed in encounters with male patients (OR 1.331), NRCMS (OR 1.551) and no insurance (OR 2.470), but less likely to be prescribed in longer consultations. Essential medicines were more likely to be prescribed in encounters with prescription refill (OR 1.754), chronic disease (OR 1.296) and longer consultation time, but less likely to be prescribed with NRCMS (OR 0.812). TCPMs were more likely to be prescribed in encounters with EBMS (OR 1.201) and ≥ 3 problems (OR 2.926), but less likely to be prescribed with NRCMS (OR 0.631) and no insurance (OR 0.626). Please see Table 7.

**Table 7:** Binary logistic regression analysis of factors associate with the prescribing indicators (*n* = 7910)

Encounter characteristics	Prescription of ≥3 medicines in the encounter		Prescription of generic medicines in the encounter		Prescription of antibiotics in the encounter		Prescription of injections in the encounter		Prescription of essential medicines in the encounter		Prescription of TCPMs in the encounter	
	Adjusted OR (95% CI)	P	Adjusted OR (95% CI)	P	Adjusted OR (95% CI)	P	Adjusted OR (95% CI)	P	Adjusted OR (95% CI)	P	Adjusted OR (95% CI)	P
Gender												
Female (ref)												
Male	**1.138 (1.017, 1.274)**	**0.025** ^*^	1.001 (0.830, 1.205)	0.996	1.078 (0.946, 1.228)	0.259	**1.331 (1.049, 1.688)**	**0.019** ^*^	1.058 (0.954, 1.173)	0.283	0.963 (0.872, 1.064)	0.459
Age group												
0–14 (ref)												
15–24	0.879 (0.516, 1.498)	0.636	1.369 (0.645, 2.903)	0.413	0.824 (0.512, 1.327)	0.425	0.963 (0.405, 2.292)	0.932	**1.771** **(1.097, 2.859)**	**0.019** ^*^	0.809 (0.496, 1.319)	0.395
25–44	**0.647** **(0.428, 0.979)**	**0.039** ^*^	1.065 (0.592, 1.914)	0.834	**0.608** **(0.419, 0.882)**	**0.009** ^*^	0.642 (0.314, 1.312)	0.224	1.319 (0.919, 1.893)	0.133	0.839 (0.573, 1.227)	0.365
45–59	**0.649** **(0.431, 0.977)**	**0.038** ^*^	1.039 (0.581, 1.856)	0.898	**0.479** **(0.330, 0.695)**	**0.000** ^*^	0.655 (0.323, 1.329)	0.241	1.191 (0.833, 1.702)	0.337	0.859 (0.590, 1.252)	0.429
≥ 60	**0.615** **(0.407, 0.931)**	**0.021** ^*^	1.193 (0.661, 2.153)	0.559	**0.464** **(0.317, 0.677)**	**0.000** ^*^	1.103 (0.545, 2.235)	0.785	1.321 (0.920, 1.895)	0.131	0.830 (0.568, 1.213)	0.336
Reason for encounter												
Present with at least one symptom (ref)												
Regular follow up	**0.544** **(0.430, 0.690)**	**0.000** ^*^	1.391 (0.958, 2.019)	0.082	**0.236** **(0.160, 0.348)**	**0.000** ^*^	**0.477** **(0.289, 0.785)**	**0.004** ^*^	0.960 (0.794, 1.160)	0.672	**0.222** **(0.182, 0.270)**	**0.000** ^*^
Prescription refill without other complaint	0.926 (0.805, 1.065)	0.281	**2.948** **(2.215, 3.923)**	**0.000** ^*^	**0.212** **(0.169, 0.265)**	**0.000** ^*^	**0.222** **(0.150, 0.328)**	**0.000** ^*^	**1.754** **(1.529, 2.012)**	**0.000** ^*^	**0.366** **(0.324, 0.413)**	**0.000** ^*^
Other reasons	**0.144** **(0.075, 0.274)**	**0.000** ^*^	**0.033** **(0.024, 0.045)**	**0.000** ^*^	**0.277** **(0.173, 0.443)**	**0.000** ^*^	0.767 (0.441, 1.336)	0.350	**0.096** **(0.068, 0.135)**	**0.000** ^*^	**0.042** **(0.026, 0.066)**	**0.000** ^*^
Insurance type												
PIIS (ref)												
EBMS	**1.279** **(1.099, 1.489)**	**0.002** ^*^	1.158 (0.855, 1.567))	0.343	0.960 (0.801, 1.150)	0.657	1.173 (0.808, 1.704)	0.402	0.870 (0.751, 1.008)	0.064	**1.201** **(1.045, 1.380)**	**0.010** ^*^
NRCMS	**0.865** **(0.749, 1.000)**	**0.049** ^*^	**0.553** **(0.429, 0.713)**	**0.000** ^*^	0.963 (0.816, 1.137)	0.659	**1.551** **(1.119, 2.149)**	**0.008** ^*^	**0.812** **(0.710, 0.928)**	**0.002** ^*^	**0.631** **(0.556, 0.716)**	**0.000** ^*^
Other insurance	1.092 (0.797, 1.498)	0.583	0.864 (0.484, 1.542)	0.620	0.905 (0.635, 1.290)	0.583	1.392 (0.712, 2.724)	0.334	0.831 (0.622, 1.112)	0.213	0.899 (0.675, 1.196)	0.464
No insurance	1.011 (0.757, 1.351)	0.939	**0.511** **(0.343, 0.763)**	**0.001** ^*^	0.936 (0.703, 1.246)	0.650	**2.470** **(1.510, 4.040)**	**0.000** ^*^	0.907 (0.702, 1.171)	0.452	**0.626** **(0.488, 0.803)**	**0.000** ^*^
First time visit												
No (ref)												
Yes	0.945 (0.730, 1.223)	0.666	**0.523** **(0.378, 0.723)**	**0.000** ^*^	1.048 (0.820, 1.341)	0.707	1.238 (0.798, 1.921)	0.341	0.808 (0.651, 1.003)	0.053	**0.693** **(0.557, 0.861)**	**0.001** ^*^
Contracted patient												
No (ref)												
Yes	**1.159** **(1.019, 1.320)**	**0.025** ^*^	0.989 (0.802, 1.218)	0.916	0.885 (0.766, 1.022)	0.097	0.852 (0.652, 1.114)	0.241	1.040 (0.927, 1.168)	0.502	0.994 (0.889, 1.113)	0.923
Chronic disease												
No (ref)												
Yes	0.963 (0.832, 1.116)	0.620	1.076 (0.853, 1.357)	0.536	**0.455** **(0.382, 0.542)**	**0.000** ^*^	1.215 (0.901, 1.640)	0.202	**1.296** **(1.138, 1.475)**	**0.000** ^*^	**0.449** **(0.396, 0.508)**	**0.000** ^*^
Number of problems involved												
< 3(ref)												
≥ 3	**6.753** **(5.806, 7.853)**	**0.000** ^*^	**2.074** **(1.398, 3.077)**	**0.000** ^*^	**2.875** **(2.361, 3.499)**	**0.000** ^*^	0.773 (0.522, 1.145)	0.199	**2.089** **(1.731, 2.520)**	**0.000** ^*^	**2.926** **(2.511, 3.410)**	**0.000** ^*^
Consultation time												
< 5 min (ref)												
5–10 min	**1.537** **(1.347, 1.755)**	**0.000** ^*^	**2.271** **(1.848, 2.790)**	**0.000** ^*^	0.950 (0.814, 1.110)	0.518	**0.701** **(0.536, 0.917)**	**0.009** ^*^	**1.630** **(1.447, 1.836)**	**0.000** ^*^	1.016 (0.905, 1.139)	0.792
> 10 min	0.981 (0.827, 1.163)	0.826	**2.809** **(2.127, 3.711)**	**0.000** ^*^	0.858 (0.713, 1.031)	0.102	**0.562** **(0.398, 0.792)**	**0.001** ^*^	**1.367** **(1.182, 1.579)**	**0.000** ^*^	**0.819** **(0.710, 0.945)**	**0.006** ^*^

*Abbreviation*: *TCPM* Traditional Chinese patent medicine, *PIIS* Public institution insurance scheme, *EBMS* Employee basic medical scheme, *NRCMS* New rural cooperative medical scheme, *OR* Odds ratio, *CI* Confidence interval

Encounters with missing values were excluded, 7910 encounters were analyzed; other reasons for encounter included check-up, transfusion and injection, sickness certificate, etc.

^*^Significant in bold

## Discussion

China has made a lot of efforts to promote rational use of medicines in primary care [7]. General practice clinics in CHSCs are crucial for the accessibility and rational use of medicines in the rural areas. In this study, four indicators including, median number of medicines per encounter, the percentage of generic medicines prescribed, the percentage of encounters with antibiotics prescribed and the percentage of encounters with injections prescribed showed similar or fair performance in comparison with data from the WHO or other studies. Median number of medicines per encounter in this study was 2.0, which is on the edge of the recommended value (below 2.0) by the WHO [16]. In comparison with domestic studies, the number reported was 2.94 in a systematic review on irrational use of medicines, which included 30 studies between 1993 and 2013 in China [17]. However, the study settings were different, many studies were carried out in county hospitals, different departments e.g. surgery department were included in the analysis. Two studies in Beijing investigating urban CHSCs in Haidian (1.88) and Dongcheng (1.9) Districts showed similar but even lower results than in this study [18, 19]. China had launched a series of policies to restrain poly-pharmacy in CHSCs. The zero mark-up policy was implemented in 2009, the CHSCs were required to procure medicines under government control, medicines would be sold at the procurement price. The policy was intended to reduce the cost of medicines and ultimately relieve the financial burden of patients, particularly those with low-income level [20]. Furthermore, GPs’ remuneration was disconnected with the number of medicines sold, ensuring they are not motivated to prescribe more medicines than necessary [21]. The Insurance Administrative Agency of Beijing also made tight control on the average number of medicines and prescription fee of encounters, if the GPs prescribe more medicines than the limit, financial punishment might be imposed [22]. The percentage of generic medicines prescribed was 97.0% in this study, which is lower than the ideal value (100%) recommended by the WHO [16]. And it is very close to the percentage (96.12%) from a study in 10 Chinese county hospitals in Anhui province in 2012 [23], and much higher than that (64.12%) in the village clinics of 10 western provinces of China in 2005 [24]. The National Health and Family Planning Commission of China had made clear that all of the prescription drugs should be written in generic names since 2007 [22], which is the main reason for the significant improvement, similar results (> 95%) were also found in other domestic studies [19, 25]. The percentages of encounters with antibiotics or injections prescribed were 15.1 and 3.7% in this study, which were below the recommended values (30% for antibiotics and 20% for injections) by the WHO [16]. The percentages in this study were also lower than the median percentages of antibiotic (52.6%) and injection (40.75) prescriptions in the systematic review of 30 studies between 1993 and 2013 in China [17]. But the percentages were still higher than the results from urban CHSCs in Haidian (6.92% for antibiotics and 2.62% for injections) and Dongcheng (6.8% for antibiotics and 3.3% for injections) Districts in Beijing [18, 19]. China formally implemented a regulation on the administration of antibiotics to promote the rational use of antibiotics in 2012 [8]. The National Health and Family Planning Commission also campaigned for rational use of medicines since 2013 to decrease irrational use of antibiotics and injections [26]. The national quality standard required very strictly for CHSIs to control the percentage of antibiotic prescriptions below 20%. Another study in 4 provinces of China also reported improvement in antibiotic prescriptions in primary care institutions between 2009 and 2010 after the establishment of essential medicine system [27]. Many patients use TCPMs as alternative choices for mild respiratory problems, there were two TCPMs (ganmaoqingre and qingkailing) for common cold in the most frequently prescribed ten medicines in this study. Strong policy intervention might be the main reason for the relatively low utilization of antibiotics and injections in CHSCs, however, long term monitoring should be maintained to evaluate the sustainability of rational use of antibiotics and injections.

The percentage of medicines prescribed from EML was 58.2% in this study, which is lower than the data (89.4%) in WHO’s report in 2006 [28], and is between the percentage of essential medicines prescribed (48.85%) in the county hospitals of Anhui province [23] and the percentage (67.70%) in the village clinics of 10 western provinces of China [24]. The result is also lower than the percentages in the studies investigating urban CHSCs in Haidian (69.44%) and Dongcheng (84.2%) districts in Beijing [18, 19]. In WHO’s report of 2011, a median of 397 medicines were included in EML globally, 441 medicines for middle income and 1706 medicines for high income countries [2]. Since the establishment of essential medicine system in China, two versions of EML were released in 2009 and 2012 respectively. In the EML of 2012, there were 520 medicines (203 medicines were TCPMs) [29]. However, the current EML in China might not be sufficient to cover the dynamic needs of patients [30], many medicines needed by the patients in community were not included in the EML yet [31]. The availability of essential medicines in primary care institutions was also a problem reported previously, in the western province Shanxi of China, the mean availability of low-price generics (included in the EML) in primary care institutions decreased significantly from 27.4% in 2010 to 22.3% in 2012 [32]. A survey on 21 essential medicines in Beijing in 2013 showed the availability of 6 essential medicines was lower than 15%, and the availability of 7 medicines was lower in the rural areas than in the urban areas [33]. Another survey in 14 CHSCs in Beijing, also showed the availability was a problem in rural areas because of long distance for delivery, and the high demand of effective and inexpensive medicines [31]. These may explain the relatively low percentage of essential medicines prescribed in this study. The essential medicine policy alone will not be enough to promote the accessibility of medicines in the rural areas, supporting policy in terms of health insurance and medicine supply is also in need. Another aspect note-worthy is the considerable amount of TCPMs prescribed in this study. In China, many of TCPMs are widely used due to the healing effect, convenience and inexpensiveness, but the prescribing pattern of TCPMs was not frequently reported. The percentage of encounters with TCPMs prescribed was 52.7% in this study, which is lower than the percentage in a study from Sichuan province (60.0%) [15]. However, whether the TCPMs are rationally used is still a question to be answered by more evidences.

The prescribing indicators were influenced by different encounter characteristics, patients with NRCMS were less likely to be prescribed with ≥3 medicines, essential medicines and TCPMs, but were more likely to use injections in the encounter. The NRCMS was launched in 2003 and improved the access and coverage of health care in the rural areas [34], but the scheme was limited by lower reimbursement rate and less extensive EML coverage than the PIIS and EBMS [30, 35], which may explain the relatively lower results in the 3 indicators comparing with better insurance schemes. Patients with ≥3 problems were more likely to be prescribed with ≥3 medicines, antibiotics and TCPMs, which reflected the higher need from patients with multiple health problems being treated [36]. We also found antibiotics were less likely to be prescribed among older and chronic patients, which agrees with another study from Andorra by Vallano et al. [37]. Consultation length is a core patient care indicator recommended by the WHO, and is a potential indicator influencing the quality of care [38]. The overuse of antibiotics in short consultations was reported in a study [39], we found generic and essential medicines were more likely and injections were less likely to be prescribed in longer consultations, however no significant influence on antibiotic prescription was found from consultation time.

### Limitations

This study mainly used 6 WHO core prescribing indicators to investigate the prescribing patterns, however more indicators could be included in further study e.g. patient care, facility indicators and adherence to clinical guidelines [3]. A relatively short time span investigated was another limitation, which might impair the study’s capacity to show more exhaustive information on prescribing patterns. The CHSCs and participating GPs were chosen by purposive sampling, which is a type of non-probability approach. The generalizability might be impaired, but purposive sampling could be efficient and valid if appropriate criteria of selection are set up [40], and the total number of encounters in our study is much more than the number required in WHO’s guideline for a cross-sectional survey of drug use pattern. More experienced GPs (88.2%) who had been working for > 10 year were recruited in this study, it was unintentionally caused by the workforce structure of rural GPs, there is a shortage of new GPs because the rural areas are still not attractive for medical graduates [41]. However, the problem should be solved by a stratified sample which ensures a defined number of young GPs to be included in future study. Despite the limitations of this study, we used WHO indicators and an additional indictor to systematically illustrate the prescribing patterns of general practice clinics in 14 CHSCs in rural Beijing. The indicators showed very similar results with studies in urban CHSCs of Beijing, except the antibiotic and essential medicine indicators, which might be caused by the differences in health needs and population demographics between urban and rural areas. The study investigated relatively detailed information on encounter characteristics, and provided a benchmark for monitoring the prescription quality of general practice clinics of rural Beijing in the future, the results can also be used for comparisons with data from other regions and countries.

## Conclusions

The government has made strong intervention on rational use of medicines in CHSCs, most indicators in this study showed similar or fair performance in comparison with WHO and domestic reports, except the percentage of medicines prescribed from EML, which is much lower than the WHO’s data. The prescribing indicators were influenced by different encounter characteristics. However, there is an absence of national or regional data for us to draw further conclusions on the prescription quality of general practice clinics in rural Beijing. National or regional ideal values based on local situations e.g. the ideal number of medicines and percentage of essential medicines might be needed in the future [42]. This will enable us to have a better understanding of how GPs are performing in terms of prescription quality. In further study, more indicators should be used to show detailed information on prescription quality, regular monitoring on the prescription quality of general practice clinics in rural Beijing should also be maintained.

## Data Availability

The datasets used and/or analyzed during the current study are available from the corresponding author (xq6518@163.com) on reasonable request.

## Abbreviations

CHSC: Community health service center
CHSI: Community health service institution
CHSS: Community health service station
CI: Confidence interval
EBMS: Employee basic medical scheme
EML: Essential medicines list
GP: General practitioner
NRCMS: New rural cooperative medical scheme
OR: Odds ratio
PIIS: Public institution insurance scheme
TCPM: Traditional Chinese patent medicine
WHO: World Health Organization
